# Characterization of *E. coli* Isolates Producing Extended Spectrum Beta-Lactamase SHV-Variants from the Food Chain in Germany

**DOI:** 10.3390/microorganisms9091926

**Published:** 2021-09-10

**Authors:** Alexandra Irrgang, Ge Zhao, Katharina Juraschek, Annemarie Kaesbohrer, Jens A. Hammerl

**Affiliations:** 1 Department Biological Safety, German Federal Institute for Risk Assessment (Bundesinstitut für Risikobewertung, BfR), D-10589 Berlin, Germany; katharina.juraschek@bfr.bund.de (K.J.); annemarie.kaesbohrer@bfr.bund.de (A.K.); Jens-Andre.Hammerl@bfr.bund.de (J.A.H.); 2 China Animal Health and Epidemiology Center, Qingdao 266032, China; cathyge2015@126.com; 3 Institute for Veterinary Public Health, University of Veterinary Medicine, 1210 Vienna, Austria

**Keywords:** ESBL, SHV-12, SHV-2, food chain, IncX3, IncI1

## Abstract

Resistance of bacteria to 3^rd^ generation cephalosporins mediated by beta-lactamases (ESBL, pAmpC) is a public health concern. In this study, 1517 phenotypically cephalosporin-resistant *E. coli* were screened for the presence of *bla*_SHV_ genes. Respective genes were detected in 161 isolates. Majority (91%) were obtained from poultry production and meat. The SHV-12 beta-lactamase was the predominant variant (*n* = 155), while the remaining isolates exhibited SHV-2 (*n* = 4) or SHV-2a (*n* = 2). A subset of the isolates (*n* = 51) was further characterized by PCR, PFGE, or whole-genome sequencing and bioinformatics analysis. The SHV-12-producing isolates showed low phylogenetic relationships, and dissemination of the *bla*_SHV-12_ genes seemed to be mainly driven by horizontal gene transfer. In most of the isolates, *bla*_SHV-12_ was located on transferable IncX3 (~43 kb) or IncI1 (~100 kb) plasmids. On IncX3, *bla*_SHV-12_ was part of a Tn6 composite transposon located next to a Tn3 transposon, which harbored the fluoroquinolone resistance gene *qnrS1*. On IncI1 plasmids, *bla*_SHV-12_ was located on an incomplete class 1 integron as part of a Tn21 transposon. In conclusion, SHV-12 is widely distributed in German poultry production and spreads via horizontal gene transfer. Consumers are at risk by handling raw poultry meat and should take care in appropriate kitchen hygiene.

## 1. Introduction

Resistance of Enterobacteriaceae to third generation cephalosporins (3^rd^ GC) is mostly mediated by the production of extended spectrum beta-lactamases (ESBLs). Third GCs are commonly used in human medicine due to their broad-spectrum activity against gram-positive and -negative bacteria and comparatively low side effects [1]. However, cephalosporins are also approved for various therapeutic applications in veterinary medicine and applied on a constant scale, whereas the general consumption of antimicrobials in animals decreased [2]. ESBLs can be detected from samples of human, livestock and meat, and companion animals, as well as from the environment [3]. According to the “One Health” concept, the different sectors are in close contact and a multi-directional transmission of (resistant) bacteria between them will take place in the absence of strict control measures. Resistance mediated by ESBLs is mostly associated with mobile genetic elements (i.e., plasmids, integrons, transposons), which substantially enhances the spread of these determinants. Although there is frequent transmission of bacteria between the sectors, the majority of the resistant bacteria were shown to be adapted to their ecosystems and hosts. Therefore, some resistances are more associated with a specific niche and the prevailing conditions than with other ecosystems [4]. Beta-lactamases is a collective for a broad variety of different enzyme groups containing hundreds of specific variants of which some confer resistance to 3^rd^ GC [5]. According to the functional classification by Bush and Jacoby 2010, serine beta-lactamases can be assigned to (i) beta-lactamases (group 2b; substrates penicillins, early cephalosporins); (ii) broad spectrum beta-lactamases (group 2br; substrates penicillins, early cephalosporins; inhibitor resistant); (iii) ESBL (group 2be; extended spectrum cephalosporins, monobactams); and (iv) broad-spectrum ESBLs (group 2ber; resistant to clavulanic acid) [6]. TEM-1 was the first plasmid-mediated beta-lactamase detected in 1965, with hundreds of variants today. Members of the CTX-M family are currently the most frequent ESBLs [7]. Whereas CTX-M-15 is typically associated with human infections, CTX-M-1 is the most common ESBL in the food chain in Europe [8,9]. The third typical group is represented by the enzyme SHV (sulphydryl variant) encoded by *bla*_SHV_ genes. Currently, 182 different SHV variants are listed in the NCBI Reference Gene Catalog (PRJNA313047; request date: 15 January 2021). Their spectrum ranges from beta-lactamase (e.g., *bla*_SHV-4_) to broad-spectrum beta-lactamase (e.g., *bla*_SHV-1_) to ESBL (*bla*_SHV-2_) up to broad spectrum ESBL (*bla*_SHV-10_). Furthermore, SHV-38 even mediates resistance to the carbapenem imipenem [10]. The most common SHV-variant in ESBL *E. coli* from the food chain is SHV-12, and poultry seemed to represent a general reservoir for *bla*_SHV_ [11]. Based on data from the national monitoring, as well as experimental studies, chickens are an important source for *bla*_SHV_-carrying bacteria in Germany. Nevertheless, systematic and comprehensive investigations of SHV-producing *E. coli* along the food chain are rare, in contrast to studies focusing on CTX-M beta-lactamases [12,13,14,15].

In this study, SHV-producing *E. coli* from the German antimicrobial resistance monitoring programs of healthy animals and food were investigated. In-depth characterization of a subset of isolates was conducted to determine potential transmission pathways for SHV mediated resistances, their association to specific plasmid types, and to gain a better insight into the genetic environment of *bla*_SHV_.

## 2. Materials and Methods

Isolates phenotypically resistant to 3^rd^ GC obtained from the German monitoring on antimicrobial resistance (commensal *E. coli* and ESBL-/AmpC-producing *E. coli*) were investigated by multiplex real-time PCR targeting the most frequent ESBL/pAmpC genes (*bla*_TEM_, *bla*_CTX-M_, *bla*_SHV_, *bla*_CMY_) for the presence of *bla*_SHV_ [16].

In general, the annual German monitoring programs were conducted according to Commission Implementing Decision 2013/652/EU. In 2016, the monitoring programs focused on the poultry production chain, while pigs and calves were investigated in 2017. The isolates (*n* = 1517) were selected on their phenotypic resistance to 3^rd^ GC, which was determined by broth microdilution, according to CLSI guidelines (CLSI M07-A10), and MIC evaluation, according to EUCAST epidemiological cut-off values defined in 2013. Dissection of specific SHV-variants was conducted by commercial Sanger-sequencing (Eurofins Genomics, Ebersberg, Germany) of PCR products amplified using the primers SHV-F (5′-TTATCTCCCTGTTAGCCACC-3′) and SHV-R (5′-GATTTGCTGATTTCGCTCGG-3′). Fifty-one isolates were chosen for further characterization. Isolates were characterized in regard to their phylogenetic group by Multiplex PCR [17], their XbaI-macrorestriction patterns (PFGE) according to the PulseNet protocol (https://www.cdc.gov/pulsenet/pathogens/protocols.html, accessed on 8 September 2021), and their plasmid content (S1-nuclease PFGE). PGFE cluster analysis was conducted using Bionumerics (v7.6.3; Applied Maths; Sint-Martens-Latem, Belgium). Localization of *bla*_SHV_ genes on plasmids was determined for the 51 *E. coli* by S1 PFGE in combination with Southern Blotting Hybridization against a digoxigenin-labeled *bla*_SHV_ probe using a DIG Easy Hib and DIG Wash and Block Buffer Set (Roche Diagnostics; Mannheim, Germany) [18]. Plasmid typing was carried out by Southern Blot hybridization, as well, or by introducing *bla*_SHV_-carrying plasmids into competent *E. coli* DH10B cells (ElectroMAX^TM^ DH10B cells; Invitrogen ^TM^, Thermo Fisher Scientific; Schwerte, Germany) by electroporation [19]. Replicon typing of transferred plasmids was conducted using the PBRT 2.0 kit (Diatheva; Cartoceto, Italy). The transferability of the ESBL plasmids was investigated by filter-mating assays using *E. coli* J53 as a recipient [20].

Illumina short-read sequencing according to Borowiak et al. (2017) was performed for all SHV-2/SHV-2a-producing *E. coli*, as well as for a subset of 21 SHV-12 producing isolates, to gain a deeper knowledge on the genetic environment of *bla*_SHV_ [21]. Long-read sequencing (PacBio or Oxford Nanopore) was conducted for a subset of the sequenced isolates to develop reliable reference plasmid genomes from hybrid sequences. Illumina raw reads, as well as PacBio raw reads, were deposited in the NCBI database and are accessible under the BioProject PRJNA721573. Raw reads of isolate 17-AB0050 can be accessed under the BioProject PRJNA589028. Short read sequencing data were assembled using SPADES v. 3.13.1, while hybrid assemblies were carried out using Unicycler (v.044). PacBio sequences of the isolate 16-AB02442 was additionally de novo assembled using HGAP [22].

Genome sequences were analyzed with the BfR in-house pipeline Bakcharak (v.1.0.0; https://gitlab.com/bfr_bioinformatics/bakcharak, accessed on 8 September 2021) in regard to MLST, AMR genes, and plasmid identification. Virulence (associated) genes were detected using VirulenceFinder v.2.0.3 [23], and only results with >99.9 identity to reference gene were considered. SNP analysis was carried out using Bionumerics (v.9.6), as previously reported [18]. Identification of most related plasmids was done using plasmidID (https://github.com/BU-ISCIII/plasmidID, accessed on 8 September 2021). Annotation of sequences was conducted by PATRIC web resourced (https://patricbr.org, accessed on 8 September 2021) and multiple plasmid alignment was carried out using BRIG [24]. 

## 3. Results

**Figure 1:**
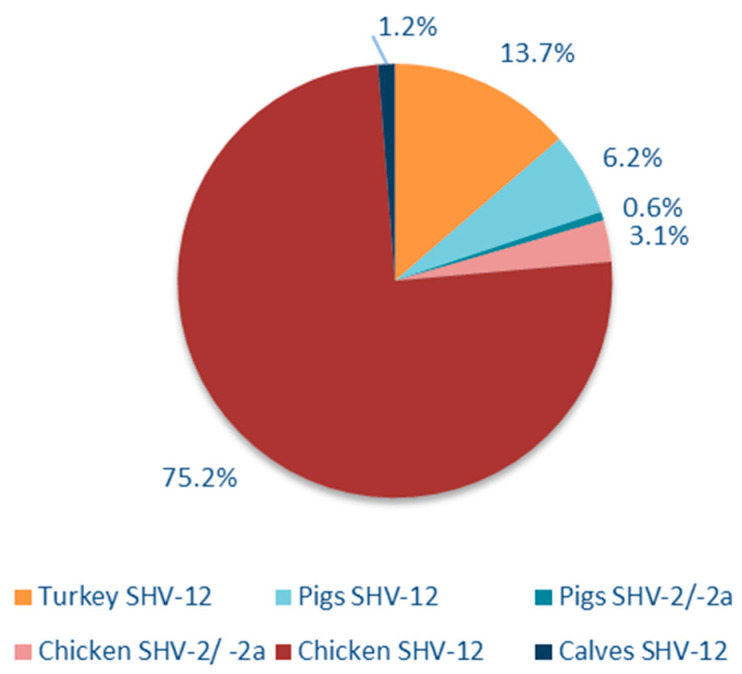
Distribution of the detected SHV-variants according to the different food production chains (*n* = 161).

In total, 1517 isolates of 3^rd^ GC-resistant *E. coli* from Germany were molecularly screened for the presence of *bla*_SHV_. One hundred and sixty-one isolates were assigned as positive for *bla*_SHV_ (Table 1), representing an overall proportion of 10.6%. The vast majority (*n* = 148) of them were obtained from the poultry production chain with an emphasis on chicken. There, a proportion of 22.2% was detected along the whole food production chain. In the turkey production chain, a lower proportion (7.4%; *n* = 22) was determined. Thirteen further isolates, originating from pigs (*n* = 11) or calves (*n* = 2), were also positive for the chosen target sequence. Subsequent typing of the prevailing SHV-variants (Figure 1) revealed that SHV-12 represents the predominant type, identified in 155 isolates (96.3%). The remaining six isolates carried *bla*_SHV-2_ (*n* = 4, 2.5 %) or *bla*_SHV-2a_ (*n* = 2, 1.2 %).

For further in-depth characterization, 51 *E. coli* were selected. The selection included all isolates from pigs and calves (*n* = 13) and 38 isolates from the poultry production chain. The SHV-2-/SHV-2a-producing *E. coli*, as well as 21 SHV-12 producing *E. coli*, were also subjected to whole-genome sequencing (WGS) analysis (Supplementary Table S1).

The phenotypic resistance profiles were considered in regard to the isolate characteristics. Among 51 investigated isolates, 29 different MIC profiles were found. In general, the isolates exhibited resistance against three to eight different antimicrobial classes (Supplementary Table S1). So, all of them were multi-drug resistant, and the vast majority (42/51) were not susceptible to ciprofloxacin. Isolates of the phylogenetic group A showed a narrow range of three to five antimicrobial classes, while *E. coli* of other phylogenetic groups showed a broader range. Overall, there was no correlation between specific resistances and phylogenetic groups or animal species.

There was a great variability found for virulence-associated genes. Between two and 25 genes (median of 14) were detected from the 27 whole-genome sequences. The three phylogenetic group A isolates harbored a maximum of four virulence associated genes, whereas isolates of the other groups exhibited a broad range of genes (Supplementary. Table S1). Further, 10 of 27 isolates were positive for *astA*. This gene encodes for the heat-stable enterotoxin 1. Most of these isolates (*n* = 6) belonged to phylogenetic group B1.

### 3.1. SVH-2-/SHV-2a-Producing E. coli

**Figure 2:**
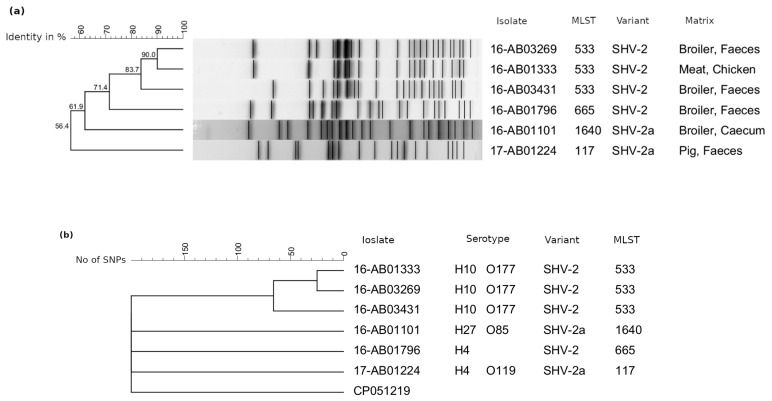
Phylogenetic relationship of SHV-2/SHV-2a producing *E. coli* from the food chain in Germany. (**a**) PFGE cluster analysis using Dice similarity coefficient and single linkage for calculation; (**b**) SNP cluster analysis calculate.

In this study, the SHV-2a variant was only detected in two isolates. One originated from broiler and one from pig. Both *E. coli* carried *bla*_SHV-2a_ on a 91 kb IncB/O plasmid but belonged to different multilocus sequence types (short STs) and phylogenetic groups (Table 2). Isolates producing SHV-2 (*n* = 4) were all obtained from the broiler production chain of different origins without obvious epidemiological linkage. Three of them belonged to ST533 and exhibited the same serotype O177:H10. XbaI-macrorestriction analysis revealed a close relationship between these isolates (Figure 2a). While the *E. coli* 16-AB01333 and 16-AB03269 were determined to be clonally related (>90%), the remaining isolates showed less similarity. The close relationship could be confirmed by single nucleotide polymorphism (SNP) analysis, although the clonality of 16-AB01333 and 16-AB03269 was based on a minimum of 23 SNPs (Figure 2b). While *bla*_SHV-2_ of the isolate 16-AB01796 was located on the chromosome, the location of the gene in ST533 isolates remains unclear. Sequence data indicates a plasmid localization, but this could not be confirmed by biological experiments (S1 Southern Blot hybridization; conjugation or transformation assays).

As only few SHV-2/2a producing isolates were available, no interpretation of the common transmission pathway can be deduced from the data. However, it is likely that *bla*_SHV-2/2a_ was disseminated through horizontal, as well as vertical, gene transfer.

### 3.2. SHV-12-Producing E. coli

In contrast to SHV-2/2a, no clonal dissemination was found for *bla*_SHV-12_ carrying isolates (Supplementary Figure S1). XbaI-PFGE analysis showed a high phylogenetic diversity among these isolates, except for 16-AB02778, 16-AB03037, and 17-AB00277. The majority of isolates was assigned to phylogenetic group A, B1, or F, which are known to represent isolates of non-clinical origin (Table 3). The spread of the ESBL determinant seems to be driven by two predominant plasmid types. A large proportion (*n* = 26/45) of the isolates harbored *bla*_SHV-12_ on ~40–45 kb (±5 kb) IncX3 plasmid. The dissemination of the IncX3 plasmids does not seem to be associated with a certain matrix or animal type. Another subset of isolates (*n* = 13/45) harbored the gene on IncI1 plasmids of 100 kb (±10 kb) in size. Plasmids of this type were mainly detected in isolates from the turkey production chain.

For three *E. coli*, the location of the *bla*_SHV-12_ gene was confirmed on a ~300 kb IncHI2 plasmid, of which one has been shown to co-express a VIM-1 carbapenemase [25].

### 3.3. Genetic Environment of bla_SHV-12_

To get a deeper insight into the genetic basis of *bla*_SHV_-carrying isolates from the German monitoring on antimicrobial resistance, short-read sequencing was performed for 21 preselected isolates (Table 3). Additionally, long-read sequencing was conducted for three isolates (IncI1 plasmid: 16-AB02442, IncX3 plasmid: 17-AB00050 and SHV-2 IncHI2 plasmid: 16-AB03269) to develop reference plasmid sequences suitable for mapping of short-read sequencing data and phylogenetic analysis.

**Figure 3:**
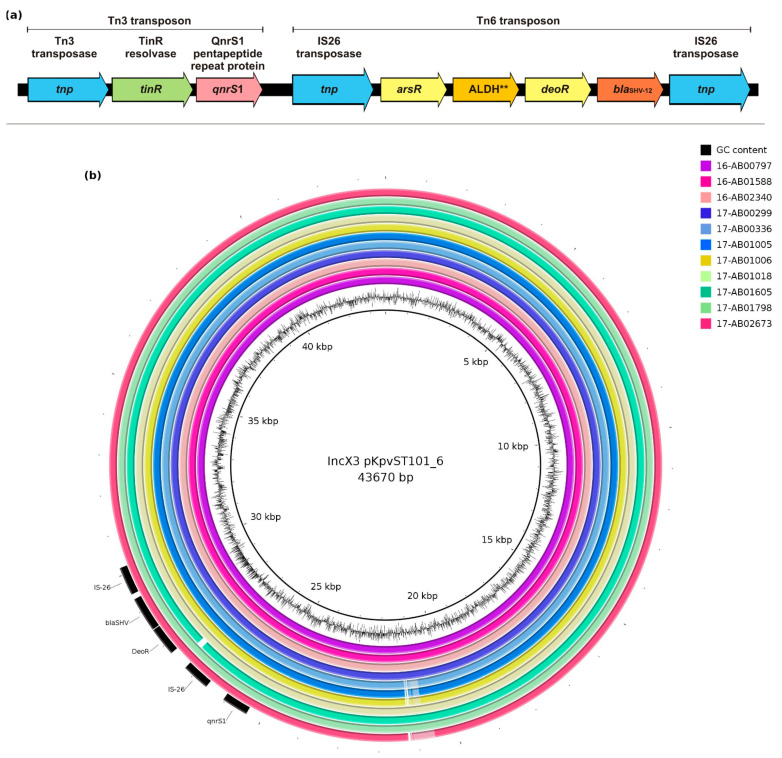
(**a**) Genetic environment of *bla*_SHV-12_ on IncX3 plasmids; ** ALDH–gene for aldehyde dehydrogenase (**b**) Mapping of Illumina short read sequences against IncX3 reference plasmid using BRIG v.0.95.

In general, assembled contigs from short-read sequencing carrying the *bla*_SHV-12_ gene are too short to provide detailed information about the genetic environment of the gene (i.e., chromosomal versus plasmidal localization) as they usually only comprised *bla*_SHV_. Reference-based mapping of the raw reads to the complete IncX3 plasmid of 17-AB00050 (available at https://www.mdpi.com/2076-2607/9/3/598/s1, accessed on 8 September 2021) showed that the complete plasmid was covered by the sequencing data of the individual isolates, except a short region of 2300 bp encoding an IS21 transposase and the ATP-binding protein IstB. Thus, a high concordance of IncX3 plasmids carrying *bla*_SHV-12_ was predicted. The genetic background of *bla*_SHV-12_ on different plasmids is illustrated in Figure 3a. In general, SHV-12 was encoded on a Tn6 composite transposon, but, due to the association of Tn6 to an IS26 transposase, short-read sequencing results were not suited for determination of the genetic basis. The repetitive sequences of the transposon commonly resulted in a deficient of the assembling software in reliable allocation of raw reads to the respective positions of the contigs. Tn6 was further associated with a Tn3 transposon encoding the acquired fluoroquinolone resistance determinant *qnrS1*. Analysis using plasmidID revealed a very high similarity of all characterized IncX3 plasmids to the *Klebsiella pneumoniae* plasmid pKpvST101_6 (CP031373; Figure 3b). This isolate was previously detected in a Chinese hospital and carries *bla*_OXA-48_ on another plasmid.

**Figure 4:**
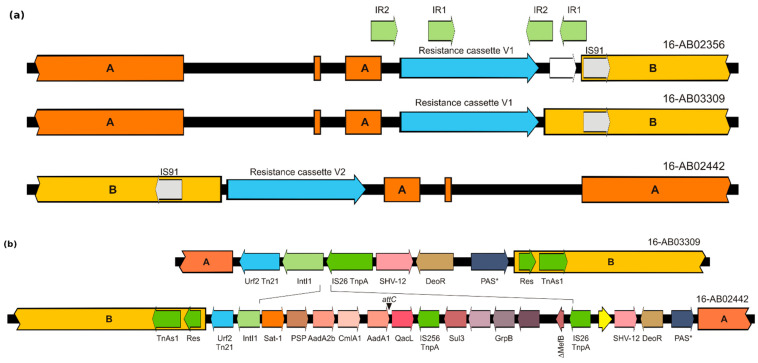
(**a**) Schematic overview of genetic environment of Tn21 derivate harboring *bla*_SHV-12_ on different IncI1 ST3 plasmids. (**b**) Detailed organization of Tn21 transposon resistance gene cassette V1 (16-AB03309) and V2 (16-AB02442). * PAS–Methyl-accepting chemotaxis sensor/transducer protein.

**Figure 5:**
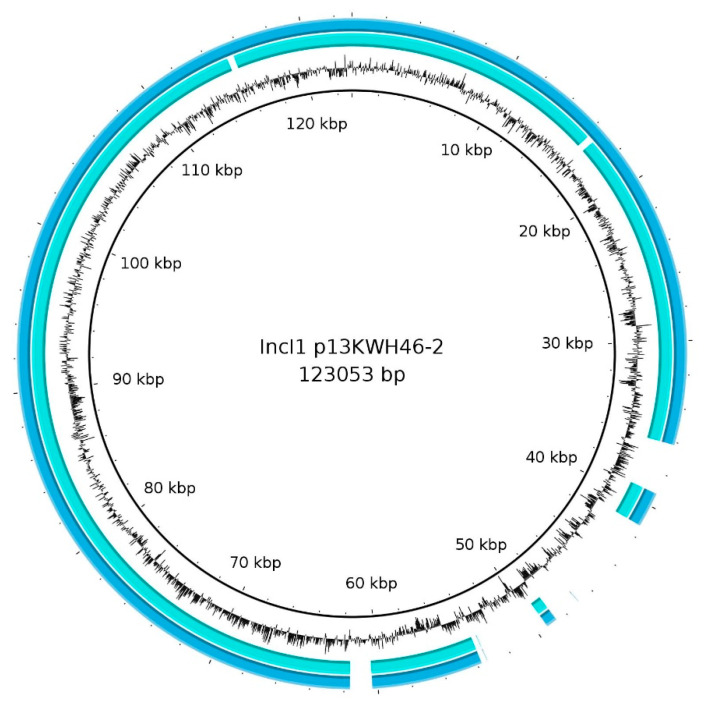
Mapping of Illumina short read sequences of two *bla*_SHV-12_-IncI1 pST3 harboring *E. coli* against reference plasmid p13KWH46-2 using BRIG v.0.95. A ~15 kb segment is missing in both isolates, encoding primarily for hypothetical proteins.

Although *bla*_SHV-12_ is also associated with IS26 on IncI1 plasmids, the genetic basis differs substantially from IncX3 plasmids. The gene is part of an incomplete class 1 integron as part of a Tn21 derivate. Based on the organization of the genes, Tn21 seemed to be inserted several times in the same region of IncI1 plasmid in different orientations (Figure 4a). The region was flanked by mobile genetic elements as different transposases and recombinases and might be a hotspot for integration or homologous recombination. The majority of the isolates exhibited an integrase with additional gene cassettes forming an atypical class 1 integron (*intI1-estX-psp-aadA2b-cmlA1-aadA1-qacL-IS256-sul3*), followed by *bla*_SHV-12_ as part of a transposable element (shown for 16-AB02442; Figure 4b). In 16-AB03309, a substantial part of the integron was not present (Figure 4b). A similar plasmid organization was detected for 16-AB02356. However, in this isolate, the serine recombinase and TnAs1 transposase were also absent. The typical Tn21 mercury (*mer*) operon could not be detected in any of the IncI1 sequences. Two different pMLSTs (ST3 and ST26) were detected, suggesting the presence of a similar variable region of multi-drug resistances in different plasmid backbones. This was supported by further analysis with plasmidID showing different possible reference plasmids for the two pMLSTs. The IncI1 ST3 plasmids showed greatest similarity to *E. coli* plasmid p13KWH46-2 (Acc.-No. CP019252) (IncI1, ST3), whereas IncI1 ST26 (CC-2) plasmids showed highest similarity to *Salmonella* Typhimurium plasmid TY474p2 (Acc.-No. NC_017675) (IncI1 ST27 CC-2). Nevertheless, alignment of p13KWH46-2 and TY474p2 showed high similarities between these two IncI1 plasmids. Both plasmids did not harbor any resistance genes. The relationship of p13KWH46-2 and IncI1 ST3 plasmids is shown in Figure 5. This reference plasmid harbored an additional ~15 kb segment, primarily encoding hypothetical proteins.

## 4. Discussion

Based on the prevailing data among all ESBL-producing isolates of the food chain in Germany in 2016/2017, *bla*_SHV_-carrying isolates are mainly associated with broilers (22%) and turkey (7.5%). This is in concordance to reports from the Netherlands, where SHV-production was also primary attributed to isolates from the poultry production chain [26]. In contrast, SHV-12-production was only confirmed for 13 isolates from pigs and veal (feces or at slaughter) but not from meat. This might represent the low prevalence of ESBL *E. coli* from pork and veal (5.5% and 4.4%, respectively) in comparison to the high ESBL occurrence among pigs at slaughter (47%) and veal calves (68%) [27]. Further, pig and calf associated *E. coli* isolates predominantly harbored *bla*_CTX-M-1_ as an ESBL determinant [11]. All characterized isolates showed multidrug resistance, which enables co-selection by antimicrobial use in meat production. High co-resistance to ciprofloxacin mirrors the wide use of fluoroquinolones, especially in poultry production [28].

The heat-stable enterotoxin gene *astA* was found in ten of 26 sequenced isolates. Enterotoxin AST1 is associated with diarrheal illness and was also detected in some enteroaggregative *E. coli* [29,30]. It can be detected in isolates from humans and animals, while its impact on the disease is still discussed [31,32]. As all samples originated from non-clinical animals, the toxin did not seem to have obvious influence on animal health. However, we have no definite information on the health status of the animals. Up to now, the pathogenicity of these strains for humans or the risk for consumers cannot be estimated. 

The heat-stable enterotoxin gene *astA* was mainly found in isolates of the phylogenetic group B1, which is uncommon for this group. There is the general assumption that A and B1 are associated with high resistance and low virulence in contrast to B2 and D [33]. This could not be confirmed based on the results of this study. Isolates of phylogenetic group A showed lowest resistance according to the number of antimicrobial classes, as well as only low numbers of virulence genes. Other isolates of phylogenetic group C, which was formerly integrated in phylogenetic group A, harbored 14–25 virulence-associated genes. 

Only few isolates producing SHV-2/SHV-2a were detected here. Both variants (SHV-2/2a) differ in one amino acid. In Europe, SHV-12 is the predominant SHV variant associated with poultry whereas SHV-2/2a has only a small share [11]. This is in contrast to Asian and American investigations [34,35]. In a Canadian study, animals (especially chicken) and food samples (chicken meat) were investigated. Therewith, all 20 SHV-producing Enterobacteria were positive for SHV-2/2a [34]. In their study, the SHV genes are mainly located on IncI1 plasmids but are not associated with IncB/O or IncX1, as found in Germany. SHV-2/2a variants were also sporadically detected within human clinical samples but seem to be mainly associated with *Klebsiella pneumoniae* [36,37,38].

The majority of the investigated isolates carried *bla*_SHV-12_ on 40-50 kb IncX3 plasmids. The increasing occurrence of IncX3 plasmids was previously described [39]. This plasmid type exhibits a highly conserved backbone with a variable region acting as a hotspot for integration and excision of mobile genetic elements. Plasmid variants bearing *bla*_SHV-12_ incorporated a Tn6 composite transposons in association with *qnrS1*, which seems to be very successful during environmental selection as they had replaced the typical IncI1 plasmids [26,40]. Thus, it can be assumed that these plasmids might be more stable and, presumably, without any further fitness costs for their bacterial hosts [39]. IncX3 plasmids are reported to be highly transmissible and replicate well in bacterial isolates from different animal species. So, in terms of international trade and traveling, it is worrying that IncX3 plasmids are highly associated with carbapenem-producing Enterobacteriaceae from human and from retail meat in South East Asia and the United Arab Emirates [28,29].

IncI1 plasmids are frequently reported as carriers of ESBL genes and often represent sizes of 100 kb [41]. A predominant plasmid MLST is pST3. This type was also reported for CTX-M-1-producing *E. coli* from food in Germany [15]. Interestingly, whereas *bla*_CTX-M-1_ was shown to be inserted into the shufflon region, *bla*_SHV-12_ was associated with an atypical class 1 integron containing multidrug-resistance cassettes with a close relationship to structures described by Alonso et al. 2017 [40]. Although they found pST26 IncI plasmids in isolates from different hosts, all IncI1 ST26 harboring isolates from our study were detected from poultry. Further, the isolates 16-AB01700 (turkey, cecum) and 16-AB03438 (turkey, meat) showed comparable characteristics (ST428; phylogenetic group B2, resistome), suggesting that a potential transmission from animal to food might have taken place during slaughter. As similar plasmids were also found in humans, a transmission between humans and animals seems to be likely, underlining hygiene importance in all stages from food production [40].

## 5. Conclusions

In Germany, SHV beta-lactamases were mainly detected from poultry production and meat. SHV-12 was the predominant variant found in this study and associated with IncX3 and IncI1 plasmid dissemination. Although cephalosporins are not applied in poultry production, co-selection might occur through further harbored antimicrobial resistance genes. No differences could be detected between their proportion in animal and meat samples. There is an undeniable risk for consumers for colonization with ESBL *E. coli* during handling of raw poultry meat with insufficient kitchen hygiene or consumption of contaminated products. Efforts are needed to reduce colonization of chicken and to improve slaughtering techniques for minimizing cross contamination of poultry meat.

## Figures and Tables

**Table 1:** Results of real-time PCR investigations of 3^rd^ GC-resistant *E. coli* from the German monitoring on antimicrobial resistance (commensal *E. coli* and ESBL-/AmpC-producing *E. coli*) on the occurrence of *bla*_SHV_. #–number of.

Year	Matrix		# Isolates Investigated		# *bla*_SHV_ Positive		Ratio in %	
2016	Broiler production total		567		126		22.2	
		Broiler, feces		166		33		19.9
		Broiler, cecum		184		42		22.8
		Broiler, skin		5		2		40.0
		Chicken, meat		212		49		23.1
2016	Turkey production chain total		296		22		7.4	
		Turkey, cecum		119		9		7.6
		Turkey, meat		177		13		7.3
2017	Pork production chain total		344		11		3.2	
		Fattening pigs, feces		325		11		3.4
		Pork		19		0		0.0
2017	Beef production chain total		250		2		0.8	
		Veal calves, feces		236		2		0.8
		Beef		14		0		0.0
2016/ 2017	other samples	Game, meat and feces (wild boar, deer, roe deer); vegetables, sprouts	60		0		0.0	
	Total		1517		161		10.6	

**Table 2:** Characteristics of SHV-2/2a-producing *E. coli* from the food production chain in Germany 2016/2017.

Isolate	Origin	SHV Variant and Localization (Size)		Inc. Group	Phylogenetic Group	MLST
16-AB01333	Meat, chicken	SHV-2	n.d.	n.d.	B1	533
16-AB01796	Broiler, feces	SHV-2	Chromosome		A	665
16-AB03269	Broiler, feces	SHV-2	n.d.	n.d.	B1	533
16-AB03431	Broiler, feces	SHV-2	n.d.	n.d.	B1	533
16-AB01101	Broiler, cecum	SHV-2a	Plasmid (87 kb)	B/O	E	1640
17-AB01224	Pig, feces	SHV-2a	Plasmid (91 kb)	B/O	F	117

Abbreviation: n.d., not determined.

**Table 3:** Main characteristics of SHV-12-producing *E. coli*. In general, the plasmid size was determined by S1 PFGE. Plasmid sizes of indicated isolates (*) were obtained from sequencing data. For all isolates with assigned MLST, sequencing data are available at NCBI under the BioProject PRJNA721573.

Isolate	Origin	SHV Variant	SHV Plasmid Size and Inc Group		Phylogenetic Group	MLST
17-AB02384 *	Pig, feces	SHV-12	298 kb	HI2	B1	7593
17-AB01032	Pig, feces	SHV-12	308 kb	HI2	B1	n.a.
17-AB01030	Pig, feces	SHV-12	295 kb	HI2	C	410
16-AB00888	Turkey, meat	SHV-12	93 kb	IncI1	A	n.a.
16-AB00970	Turkey, cecum	SHV-12	100 kb	IncI1	F	n.a.
16-AB01461	Turkey, cecum	SHV-12	104 kb	IncI1	D	n.a.
16-AB01700	Turkey, cecum	SHV-12	97 kb	IncI1, ST26	B2	428
16-AB02356	Turkey, cecum	SHV-12	84 kb	IncI1, ST3	B1	162
16-AB03339	Broiler, cecum	SHV-12	100 kb *	IncI1	B1	n.a.
16-AB02442 *	Turkey, meat	SHV-12	110 kb *	IncI1, ST3	D	38
16-AB03438 *	Turkey, meat	SHV-12	105 kb 218 Kb	IncI1, ST26 IncFIB/FIC	B2	428
16-AB03529	Turkey, meat	SHV-12	107 kb	IncI1, ST26	E	57
16-AB03530	Turkey, meat	SHV-12	104 kb	IncI1	F	n.a.
16-AB03534	Turkey, meat	SHV-12	28 kb 100 kb	n.a. IncI1	A	n.a.
16-AB03309 *	Broiler, cecum	SHV-12	91 kb	IncI1, ST3	B1	1196
17-AB01138	Calves, feces	SHV-12	90 Kb	IncI1	B1	n.a.
17-AB01735	Pig, feces	SHV-12	106 kb	n.t.	A1	1060
16-AB00677	Turkey, cecum	SHV-12	39 kb	IncX3	F	n.a.
16-AB00797	Broiler, cecum	SHV-12	39 kb	IncX3	A	10
16-AB01024	Turkey, meat	SHV-12	41 kb	IncX3	F	n.a.
16-AB01389	Broiler, cecum	SHV-12	41 kb	IncX3	A	n.a.
16-AB01588	Broiler, cecum	SHV-12	43 kb	IncX3	F	117
16-AB02021	Turkey, meat	SHV-12	40 kb	IncX3	C	n.a.
16-AB02026	Turkey, cecum	SHV-12	42 kb	IncX3	B1	n.a.
16-AB02340	Turkey, cecum	SHV-12	42 kb	IncX3	B1	9046
16-AB02352	Broiler, cecum	SHV-12	44 kb	IncX3	E	n.a.
16-AB02541	Broiler, cecum	SHV-12	43 kb	IncX3	A	n.a.
16-AB02638	Turkey, cecum	SHV-12	38 kb	IncX3	B2	n.a.
17-AB02673 *	Pig, feces	SHV-12	37 kb 43 kb	IncN IncX3	C	2230
16-AB02778	Broiler, cecum	SHV-12	43 kb	IncX3	F	n.a.
16-AB03037	Broiler, cecum	SHV-12	44 kb	IncX3	F	n.a.
16-AB03425	Turkey, meat	SHV-12	46 kb	IncX3	F	n.a.
16-AB03444	Turkey, meat	SHV-12	47 kb	IncX3	D	n.a.
16-AB03515	Turkey, meat	SHV-12	61 kb	IncX3	A	n.a.
17-AB00299	Broiler, cecum	SHV-12	41 kb	IncX3	F	117
17-AB00308	Broiler, cecum	SHV-12	45 kb	IncX3	A	n.a.
17-AB00336	Turkey, cecum	SHV-12	220 kb	IncX3	F	117
17-AB01005	Pig, feces	SHV-12	39 kb	IncX3	A	1244
17-AB01006	Pig, feces	SHV-12	40 kb	IncX3	A	10
17-AB01018	Pig, feces	SHV-12	40 kb	IncX3	C	88
17-AB01605	Pig, feces	SHV-12	42 kb	IncX3	B1	n.a.
17-AB01798	Pig, feces	SHV-12	42 kb	IncX3	B1	641
17-AB02071	Calves, feces	SHV-12	41 kb	IncX3	B1	58
16-AB02401 *	Turkey, meat	SHV-12	37 kb	X1	E	n.t.
16-AB03659	Turkey, meat	SHV-12	30 kb	X1	F	n.a.

n.t.—not typable; n.a.—not analyzed.

## Data Availability

Illumina raw reads were deposited in the NCBI database under the BioProject PRJNA721573.

## Supplementary Materials

The following are available online at https://www.mdpi.com/article/10.3390/microorganisms9091926/s1, Figure S1: Phylogenetic analysis of SHV-12 producing *E. coli* from the food chain in Germany based on XbaI PFGE. Table S1: Phenotypic resistance of SHV-producing *E. coli* from the food chain in Germany, resistance genes and numbers of virulence associated resistance genes detected by whole genome sequencing.
