# Supplementary figures and images for: Characterization of E. coli Isolates Producing Extended Spectrum Beta-Lactamase SHV-Variants from the Food Chain in Germany
Source: Microorganisms. 2021 Sep 10;9(9):1926. doi: 10.3390/microorganisms9091926 (PMC8471341; doi:10.3390/microorganisms9091926)

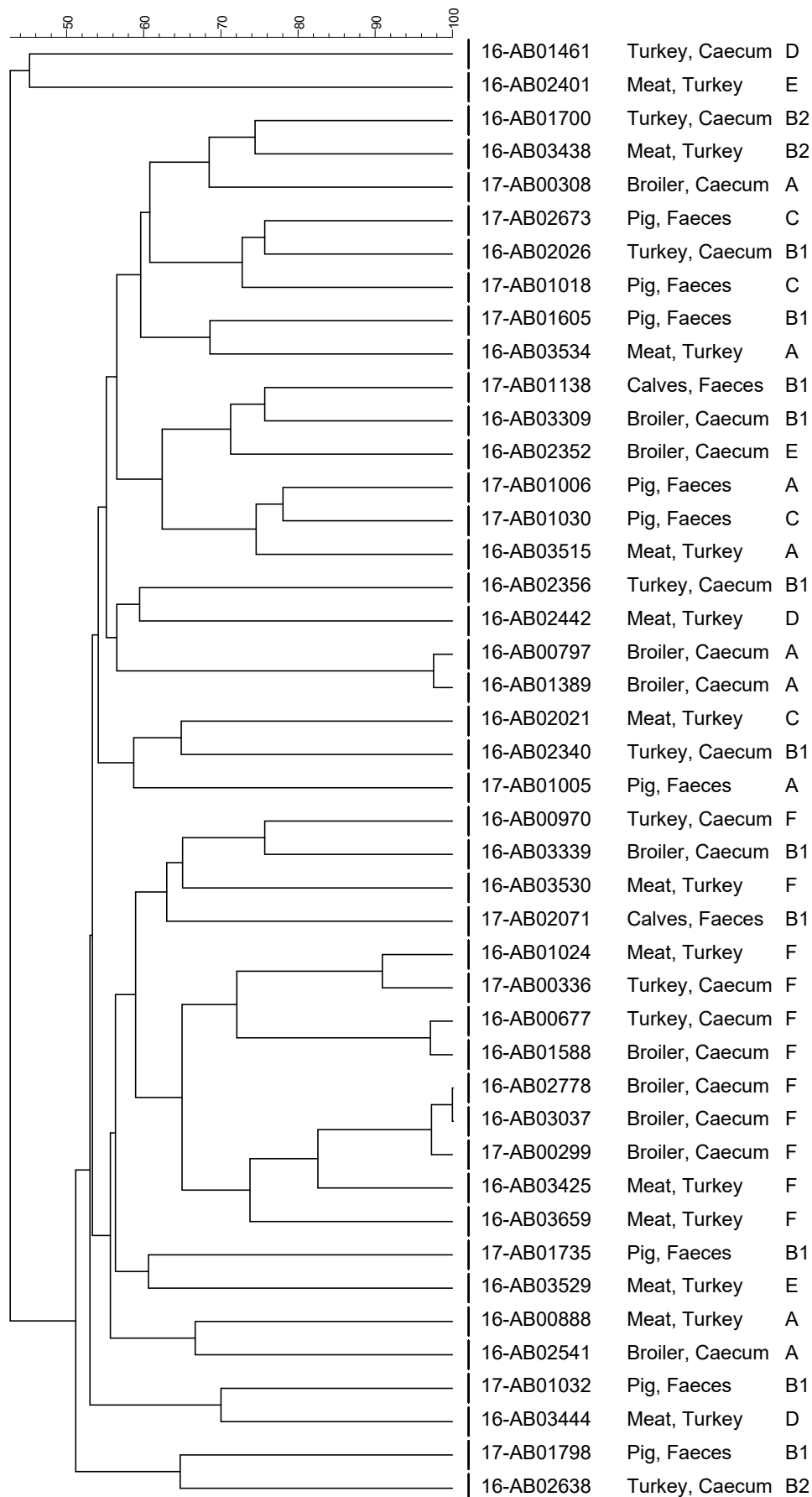

Supplement: Supplementary file 1 [file microorganisms-09-01926-s001.zip › FigS1Phylogenetic tree_Xba_SHV-12.pdf]
